# How Can Providers Better Address Recovery Needs for Geriatric TBI Survivors?

**DOI:** 10.1093/geroni/igab046.346

**Published:** 2021-12-17

**Authors:** Mia Vogel, Wonkyung Jung, Hilaire Thompson

**Affiliations:** 1 Washington University in St. Louis, St. Louis, Missouri, United States; 2 University of Washington, Seattle, Washington, United States

## Abstract

Older adults are at increased risk for poorer recovery from traumatic brain injury (TBI), incidence of pre-injury frailty, and comorbidity compared to younger adults. In this study, a longitudinal multiple case study approach was taken to describe gaps in health service delivery identified by older adults following mild-moderate TBI. Participants were interviewed at 1 week, and 1, 3, 6, and 12 months post injury (5 times). In total, 57 interviews were conducted with 13 participants and transcribed verbatim. Codes were identified inductively to develop a codebook for guiding thematic analysis in NVIVO. Two independent investigators double coded 16 transcripts (28%) and reached consensus; remaining transcripts were allocated to the two investigators for independent coding and verified and reconciled with the other coder until consensus was reached. Many participants were happy with the care they received. However, ongoing health issues and TBI symptoms sometimes remained unaddressed. Follow-up and care planning would have been helpful for most patients as well as ways to address common balance and dizziness issues. Among some, the unwillingness to go to a provider for follow-up or adhere to medication and physical therapy exercises were issues. In some cases, providers were unable to help or understand the reason for patients’ symptoms (e.g., smell/taste changes, ear issues, etc.). Sometimes, providers did not address the issues that were of most concern to patients, or they would prescribe treatments that were not acceptable to patients. Empathetic, tailored, and patient-centered approaches are needed to improve care delivery and outcomes.

